# Embracing the Complexity of Neurodevelopmental Disorders

**DOI:** 10.3390/brainsci11111385

**Published:** 2021-10-22

**Authors:** Michele Roccella, Luigi Vetri

**Affiliations:** 1 Department of Psychology, Educational Science and Human Movement, University of Palermo, 90128 Palermo, Italy; michele.roccella@unipa.it; 2 Oasi Research Institute-IRCCS, Via Conte Ruggero 73, 94018 Troina, Italy; 3 Clinic of Child and Adolescent Neuropsychiatry, Department of Mental Health and Physical and Preventive Medicine, University of Campania “Luigi Vanvitelli”, 81100 Caserta, Italy

Neurodevelopmental disorders are a group of neuropsychiatric diseases that affect the developing brain due to a complex interaction between genetic and environmental factors. The papers included in the Special Issue “Neurological Diseases in Children” reflect the complexity of the diagnosis and treatment of these conditions, representing a real challenge for clinicians.

Neurodevelopmental disorders cover a wide range of manifestations with different degrees of severity, causing an extremely variegate spectrum of phenotype that can involve every brain function. A perturbation of the brain’s development negatively influences the neurological pathways, with implications for the functioning of reading abilities, social skills, memory and attention skills. For instance, some studies suggest that children with neurodevelopmental disorders have worse performances in regard to emotion recognition. Operto et al., in their study, demonstrate that children with specific learning disorders have difficulties in understanding facial expressions compared to a group of typically developing peers. This deficit in emotion recognition is totally independent from global intelligence, but it is related to executive functions, suggesting a possible common dysfunction between recognition performances and learning abilities [1].

In these conditions, the diagnosis plays a crucial role because the time dimension is often crucial in an organ that changes exceedingly quickly, such as the developing brain. Therefore, clinicians must exploit the susceptible windows in which we have the ability to influence plastic changes in the brain due to supportive shaping environmental forces. An early and effective diagnosis, as well as precision treatment, are of fundamental importance and should be pursued for improving the outcomes of these patients. 

In this respect, Smirni explored whether a clustering of items in a psychometric test could provide better clinical indications compared to an overall score. Her data shows that performances on Raven’s Coloured Progressive Matrices may arise from complex abilities that require perceptual or analogical reasoning. Therefore, qualitative analysis parameters provide more operative clinical suggestions than a single total score, and can play a decisive role in the assessment of the strengths and weaknesses of children with intellectual disabilities [2].

A good practice in the management of neurological diseases in children is the continuous verification of the accuracy of the diagnosis and of the appropriateness of the chosen treatments. Benedetto et al. evaluated the one-year diagnostic stability of autism spectrum disorder diagnosis in a clinical sample of young children who had received an early diagnosis. The authors demonstrated that the majority of children diagnosed with autism spectrum disorder continued to show autistic symptoms at a one-year follow-up evaluation. Nevertheless, a significant percentage of subjects no longer met the criteria for autism spectrum disorder but continued to show neurodevelopmental deficits in one or more areas, highlighting a high sensibility but a moderate stability of autism early diagnosis [3].

Another interesting characteristic of neurodevelopmental disorders is that the co-occurrence of two or more different disorders represent the norm rather than an exception. Among the comorbidities in neurodevelopmental disorders, the association between autism spectrum disorder and migraine remains nearly unexplored. Vetri, in his review, analyzes the few studies found in the literature that investigate this comorbidity, and reports the common pathophysiological changes, altered immune response, cortical disorganization, dysfunctional gut–brain axis and shared susceptibility genes. The results of the literature review show a high rate of migrainous symptomatology in people with ASD, although the frequent altered pain sensitivity could distort their perception of headaches; moreover, the social dimension of pain could be impaired, causing difficulties in reporting pain [4].

One of the most debated topics in the field of research of neurodevelopmental disorders remains the understanding of the role of different genetic and environmental factors that contribute to the etiopathogenesis of these diseases and the development of more effective treatments. Operto et al., in this area, suggest limiting the use of digital devices by children during the first 3 years of life, and they encourage social interaction to support the learning of language and communication skills, because their data show lower mimic-gestural skills and lower language skills in children with longer times of exposure to digital devices [5].

Di Rosa et al. face, in their article, the challenge of the treatment of neonatal status epilepticus, reporting three cases successfully treated with hydrocortisone after the failure of conventional first- and second-line antiepileptic therapies [6]. The authors stress the importance of considering the role of the modulation of the immune response and brain inflammation in intractable epilepsy, and suggest considering nonconventional therapies such as steroidal anti-inflammatory drugs in the treatment of neonatal status epilepticus.

Finally, an interesting whole-exome sequencing case report describes an Italian family with familial hemiplegic migraine in which a heterozygous ATP1A4 mutation was identified in the absence of mutations in the genes known to be associated with familial hemiplegic migraine [7]. Four family members presented at least two migraine attacks preceded by visual symptoms and transient unilateral weakness and paresthesia. Moreover, a further 2-year follow-up showed a moderate increase in migraine attacks, which significantly improved with a low, daily dose of carbamazepine.

In summary, the articles in this Special Issue, “Neurological Diseases in Children”, embrace different aspects of neurodevelopmental disorders, shed new light on the understanding of these complex disorders and raise several new questions reflecting the imperative need for further large-scale studies in this field.
